# Orphans in Syria and Iraq Juggling Balls: Wars, COVID-19, and the NGO’s financial crisis

**DOI:** 10.1080/17482631.2023.2170010

**Published:** 2023-02-06

**Authors:** Wahiba Abu-Ras, Anas Ashraf AbuLaban, Sabreen Talat AlQaisi, Mohammed T. H. AlQaisi, Eliza Decker

**Affiliations:** a Adelphi School of Social Work, Garden City, NY, United States; b Freelance Researcher Conflict Management and Humanitarian Studies, Doha, Qatar; c Field Coordinator, Qatar Charity, Doha, Qatar; d Research Associate Orphans Project Adelphi School of Social Work, Doha, Qatar; e MSW Candidate Adelphi School of Social Work, Garden City, NY, United States of America

**Keywords:** Orphans, COVID-19, NGO crisisrisis, mental health, Wars, trauma

## Abstract

The COVID-19 pandemic’s impact varies between and within nations, causing new forms of inequality. Refugee and orphan children in conflicted areas are more likely to suffer due to poverty, vulnerability, and limited access to essential services including reduction in donor funding. This qualitative study is the first to assess the effects of the COVID-19 pandemic and the financial crisis on Iraqi and Syrian orphaned children and their mothers. The Modified Grounded Theory was used for the preliminary analysis to expand the range of themes. This study has identified five major themes: financial crisis, low educational attainment, child labour, mental health issues, violence, and social problems. The impact of COVID-19 children and adolescents’ mental health is of great concern. These multiple crises may significantly impact orphan children and adolescents’ cognitive, mental health, and physical development. It is critical to address mental health issues during the current crisis and to plan for possible future pandemics and their intersecting outcomes. A Holistic approach requires providers to be aware of their clients’ intersecting circumstances and needs using a range of lenses, including the person in the environment, family dynamics, culture, politics, and structural challenges. Offering food, medical supplies, and housing are considered basic needs.

The Coronavirus (COVID-19) pandemic has been one of the most devastating crises in recent memory. Community and individual efforts have been made to slow or stop the spread of this disease, but it continues to claim thousands of lives, and infection rates are increasing daily (S. D. Hillis et al., 2021). As a result of this pandemic, numerous mental illnesses and traumas have also been reported (de Matos Brasil et al., 2022; Waterfield et al., 2021).

The virus continues to be fought by governments and countries around the world. While the pandemic unfolds, only limited progress has been made in stopping the spread of the virus. Finding an effective vaccine or treatment remains challenging for many people and countries. As an example, refugees or persons living in war zones may not have access to vaccines. Similarly, vaccines may only be effective with access to other essential resources and services, such as adequate medical care, employment, food supply, and shelter. During this period of uncertainty, people face various challenges, such as distance learning, education, and unemployment, which affect their ability to access vital services and maintain their health and well-being (Drenovska et al., 2021). Telephones, computers, and access to the Internet have become essential tools for keeping jobs, continuing education, and providing services (Fana et al., 2020; Popyk, 2020). The majority of refugees, however, do not have access to these essentials, and as a result, many have been adversely affected by this pandemic.

Moreover, COVID-19‘s pandemic measures focused mainly on prevention, leaving behind thousands of orphans and refugees who suffered adverse consequences, including poverty. Orphans and refugees are more prone to infection and economic hardship and have fewer means to cope with income loss, trauma, and illness. Furthermore, they are less likely to receive the necessary care when infected. COVID-19 illustrates this inequality, as many refugees and orphans in the Middle East and North Africa (MENA) region have a lower survival rate than others in more developed countries (S. D. Hillis et al., 2021).

Before COVID-19, the MENA region faced many challenges, including social and political instability, a financial crisis, a high unemployment rate among young people and women in particular, and heightened political tensions across several nations (Awdeh & Hamadi, 2019; Bilgin & Kilicarslan, 2008; Elsherif, 2016; Fakih et al., 2020; Gardner, 2003; Ihle et al., 2019; Khondker, 2019; Rabiei, 2020; Sekrafi & Sghaier, 2016; Sofuoğlu & Ay, 2020; Waha et al., 2017).

Due to the freezing of funding by many donor countries, the financial situation of many NGOs degraded significantly during COVID-19, further worsening orphans’ and refugees’ conditions (Bashier et al., 2021; Olimat, 2022; Wilke et al., 2020). There are no recent studies on the effects of the COVID-19 pandemic on orphaned and refugee children in the MENA region, so this study will be the first to look at two large orphan populations from Iraq and Syria.

## The orphan refugees: caught between COVID-19 and the financial crisis

In the wake of the COVID-19 pandemic, much attention has been paid to death tolls and infected cases. UNICEF defines an orphan as “anyone aged 0–17 who has lost one parent”. Of more than 153 million orphans worldwide, 116 still have a living parent, while another 14 million have lost both parents. Sub-Saharan Africa is home to about 9 million children (UNICEF, 2021). Yet, little research has been conducted regarding COVID-19‘s impact on Iraqi and Syrian orphan children in the MENA region. Despite COVID-19 primarily affecting the elderly and people with pre-existing medical conditions, children are affected by contracting contract COVID-19 and spreading it to others (CDC, 2021b). The Center for Disease Control and Prevention (CDC, 2021a)estimates that cumulative rates of COVID-19 for children ages 5–17 were comparable to those for adults ages 18–49 and higher than those for adults ages 50 and older.. UNICEF (2020a) reported that 32% of children show signs of pneumonia to caregivers, and unreported cases are hard to track. Hill et al., (S. Hillis et al., 2021a, 2021b) found that 1,134,000 children experienced the death of a primary caregiver, including a parent. Orphans and refugees living in war zones have also experienced the loss of their parents. Children in the MENA region are more likely to contract COVID-19 because they are more prone to infection and have fewer resources to cope with war, injury, and illness. (2021)) referred to orphan children as “the hidden pandemic of COVID-19.”

Orphans in the MENA region are mainly sponsored by many NGOs (Szalai, 2019). The freezing of funds made by donor countries during COVID-19 caused most NGOs to experience a deteriorating financial situation (Wilke et al., 2020). The receiving countries also restricted NGOs to limit outbreaks, causing the loss of 11 million jobs in the MENA region and affecting over 60 million children living in poverty (UNICEF, 2021). While UNICEF provided new social assistance to 13 million households, many orphans still lack access to services, further aggravating their pre-existing vulnerabilities (UNICEF, 2021). In addition, most basic orphan care programmes have been stopped, including development and rehabilitation, empowerment, mental health, medical care, and education (Wilke et al., 2020). Therefore, many children in the MENA region face poor health, especially those in conflicted areas, such as Syrian and Iraqi orphans.

MENA is home to more than 61 million children living in war-torn countries. Conflicts and violence affect one in three children, and war is becoming a “new normal” for millions of children (UNICEF, 2017). Syrian and Iraqi children are amidst a refugee crisis that is the fastest-growing in history. Around two million Iraqis have been displaced, and another two million have fled to neighbouring countries, with 66,760 living in refugee camps in Jordan (UNHCR, 2021). In addition, about 1 million Syrian children became orphans (Nar, 2021). As of 15 November 2021, the MENA region reported 14,815,082 infected cases and 266,456 death cases. During the second week of November 2021, In Syria, the number of COVID-19 cases rose from 2.42% to 4.72%, indicating that more people are infected with the disease (Worldometers, 2023). These figures demonstrate a need to examine COVID-19‘s impact on orphans in the MENA region and how it affected other aspects of their lives.

### The epidemic and isolation crises

Developing countries risk a pandemic due to their worn health systems, lack of personnel and equipment, poverty, and deprivation (El-Ramady et al., 2021; Oppenheim et al., 2019; Oshitani et al., 2008). When pandemics occur, some individuals ignore or underreport the infection rather than commit to a long period of isolation, especially if the existing isolation conditions are unhealthy (Peterman et al., 2020). The COVID-19 crisis has kept 1.38 billion children away from school, group activities, team sports, and stadium events. During this time, mothers and caregivers were expected to be responsible for keeping children safe and occupied at home, further stressing their mental health. Such a situation leads to increased social and family violence and pressure on parents and caregivers (Cluver et al., 2020).

Orphans during crises and disasters at-risk or vulnerable populations include “children, racial and ethnic minorities, the elderly, women, persons with disabilities, and special needs” (Peek et al., 2017). However, orphans are among the most vulnerable children due to the loss of parental care and support. In times of crisis and disaster, children’s healthy development depends on the quality of care and support from caregivers, institutions, and social safety networks (Araújo et al., 2020). In such situations, children often endure psychological problems, lack of education, and difficulty accessing essential commodities and services due to the imbalance experienced by their families and social organizations (Peek, 2008). Orphans often face more significant challenges than other children due to a lower chance of attending school, less access to health care and food, and a lack of psychosocial support, making them vulnerable to exploitation and abuse (De Witt & Marike, 2007). Some are also forced to assume responsibility for their single parent (Foster et al., 2002). Due to the lack of studies examining previous epidemics’ effects on orphans, studies on parents infected by HIV can be used to illustrate the scope of the impact orphans may experience. For example, in sub-Saharan Africa, orphans face malnutrition, limited access to education, economic hardship, exploitation, and abuse (De Wagt et al., 2005). In Lesotho (Tanga, 2013), orphans drop out due to limited financial resources. In Uganda, only 29% of the 15- to 19-year-olds whose parents died from HIV were still in school, 25% dropped out temporarily, and 45% left altogether (De Wagt et al., 2005). During HIV’s spread in Tanzania, Zambia, and Zimbabwe, orphans’ increased malnutrition rates made them more prone to stunted growth and underweight (Tanga, 2013). Several orphans turned from care recipients to caregivers due to declining health services, reduced income among the affected families (Foster et al., 2002), and the reduced quality of care (De Wagt et al., 2005). As HIV spread in Rwanda, orphans had fewer opportunities to receive a formal education, a higher chance of going to work early, and were less likely to receive vaccinations (Siaens et al., 2003). Generally, orphans at disease and crisis centres suffer from malnutrition, poor health, school dropout, and violence, resulting in splendid isolation. (Shetty et al., 2003) Those in rural areas usually suffer more than their urban counterparts. Literature also suggests that caregivers become more responsible for providing orphans with education, protection, and health maintenance during epidemics (De Witt & Marike, 2007).

### The humanitarian situation in Iraq and Syria

Given the absence of national statistical surveys in Syria and Iraq, it is difficult to quantify the number of orphans and identify their most important problems; however, the humanitarian situation in both countries is clearly among the worst in the world (Nar, 2020).

In recent years, Iraq has experienced instability, unrest, and cyclical violence. The number of Iraqi orphans is estimated to be five million (5% of all orphans worldwide), and nearly the same number of young people live in poverty (Human Rights Report, 2021). Over 4.1 million people—half of them children—need humanitarian assistance. In Turkey alone, there are more than 1.2 million Syrian orphans, including abandoned children (World Bank Group, 2021, June 22). Most areas suffer from violence, retaliation, and lack of access to essential services like water, health care, and education. Ten percent of primary school-aged girls do not attend school, compared to seven percent of boys. The poorest children will likely die before age five (UNICEF, 2020b). In Iraq, 58% of orphans and other vulnerable groups attend school; 8% of primary school children drop out, and 15% die from pneumonia (UNICEF, 2021). In addition, 81% of children aged 1–14 experience physical punishment and psychological abuse from their caregivers, while 5% of children aged 5–17 (e.g., economic activities).

Syria’s situation is no better. The UN (2020) estimates that 11 million Syrians need humanitarian assistance, including 4.7 million children and 1.3 million people with disabilities. More than 6 million children have been born since the Syrian crisis began. Over 3.1 million children are under five years, and 1.6 million pregnant and lactating women need food support. Children’s rights are constantly violated when explosive weapons are deployed in civilian areas that cause them harm or death. Health and education facilities have been destroyed, leading to 2.4 million children between 5 and 17 being out of school and 1.3 million at risk of dropping out or not learning (UNICEF, 2022). Worse yet, children are being recruited as fighters for military purposes, and some are as young as seven years old. Additionally, orphaned children of Islamic State fighters receive less adequate care and resources, and fewer countries are willing to repatriate them due to fear of political opposition or a threat to national security. These children are among the most affected by the pandemic (The Lancet Child & Adolescent Health, 2020).

Displaced populations, especially in the northeast and northwest of Syria, are vulnerable to infectious diseases due to unsanitary living conditions, overextended health services, and limited routine immunization coverage. UNICEF (2022) estimates that more than 6 million newborns need regular vaccinations and that some 320,000 children aged 13–59 months are not fully vaccinated. In addition, some 15.5 million people need potable water, including 6.2 million with urgent needs for food, clothes, medical assistance, and proper education. While the full scope of explosion-related contamination remains unknown, Preliminary results suggest that nearly 2,600 communities are affected, with 11.5 million people at risk—an increase of 1.3 million between May and August 2018. The escalation of hostilities has displaced more than 630,000 people in the northwest and caused widespread destruction of homes.

As the epidemic spread throughout Iraq (UNICEF, 2020), Baghdad and international organizations took limited measures to contain it, such as sterilizing camps, providing medical equipment, improving the water supply and sanitation, and raising awareness. Similarly, UNICEF conducted 13 water, sanitation, and hygiene assessments and undertook light rehabilitation activities in Syria to support quarantine and isolation centres (UNICEF, 2020). Through a partnership with the Syrian Arab Red Crescent (SARC), UNICEF trained programme implementers and provided personal protective equipment to partners throughout the health sector. At UNICEF’s request, the World Food Program agreed to include bars of soap in its monthly food distributions. Approximately 50,000 soap bars were distributed, with plans to purchase 1.6 million more; however, this covers only 10% of the sector’s total needs (UNICEF, 2020).

Syrian data is even scarcer, especially in the north and east, where the violence continues. UNICEF (2020) shows the following statistics: 77% of children under five suffer from acute respiratory infections and seek advice from a health facility or caregiver; 9% die from pneumonia, and over 2.5 million are internally displaced. Meanwhile, 71% of Syrians have a soap and water washing facility. Furthermore, 3% of Syrian women (aged 20–24) married before they turned 15, and 15% married before 18. Families also need access to technology: only 35% have access to the Internet, and only 40% have a personal computer.

Organizations Working with Orphans

NGOs provide development and relief services in Syria and Iraq, including orphan care. Most of these organizations provided packages to orphans and their families, starting with orphanages by allocating limited funds from donors who pledged for a limited time. They also offer the orphans’ mothers specialized training courses to empower them and develop their knowledge, services, and psychological intervention programmes for widows. In addition, beneficiary-directed parallel educational programmes primarily emphasize educational aspects containing training to develop children’s skills and recreational activities (Gibbons, 2005; Machado et al., 2018; Maksudyan, 2011).

There is a problem with such approaches, however. Most of their activities and projects rely on external financial support, community support, internal donors, and local volunteers to implement them. As such undertakings have only a limited capacity due to the surrounding instability, conflict, and widespread hostility, they face uncertainty in finances, a lack of specialized and qualified personnel, poor infrastructure and technology, and difficulties in managing data and coordinating work with partners.

## Methodology

This study aimed to 1) understand how the financial crisis affects NGOs’ service provision to Syrian and Iraqi orphans and 2) identify the impact of the crisis on orphans’ social, economic, health, and educational well-being.

Nineteen participants participated in the study, eight from Iraq and 11 from Syria, using qualitative methods, including Zoom interviews. The eight Iraqi participants all lived in Iraq. Four provided essential services to NGOs, and four were mothers of orphaned children. Among the Syrian participants, five were mothers residing in Turkey at the time of the interview. Six of the remaining Syrian participants worked for NGOs; one was a mother of orphaned children.

Two researchers used a semi-structured interview protocol to conduct all interviews in Arabic. They investigated the impact of COVID-19 on orphans, the services provided by NGOs, and its effects on orphan care. Among the questions were: What concerns have you and your children experienced during the COVID-19 pandemic? In what ways has COVID-19 affected you and your family’s health, mental health, education, employment, and well-being of your family? How did COVID-19 affect NGOs’ services? What are the effects of the financial crisis on these services? Additionally, we asked mothers about the quality of the services provided by NGOs to orphans and their families and whether these crises had affected the availability or termination of projects and activities. The interviews were conducted via Zoom and WhatsApp, audiotaped, transcribed, translated from Arabic to English by a certified translator, and edited for transcription errors. The Institutional Review Board of Adelphi University approved this study (#121619- Approved on 9 November 2021)

### Research design

This study utilized a Modified-Grounded Theory Approach (M-GTA) (Kinoshita, 2019, 2015), which is a modified version of Glaser and Strauss’ grounded theory approach developed by Glaser and Strauss (2017) first in 1967 and published in 1999. Through the GTA, the theory is generated from data rather than tested against hypotheses or proposed ideas. This process involves collecting and analysing data almost simultaneously. A concept of “theory saturation” was also recommended by Glaser & Strauss of the GTA when data collection does not provide new insights. The M-GTA and grounded theory approaches have essential characteristics, including theory generation based on data, empiricism, and profound interpretation. However, Kinoshita (2019) suggested that all GTA should include five elements: 1) Theory should be grounded on dada and theory is generated; 2) Data are categorized using open coding and selective coding; 3) categories emerge from data using the constant comparative methods; 4) theoretical sampling occurs as the researcher considered the following step data collection, and 5) Theoretical saturation, in which conceptual categories have sufficient substantial evidence to support them. In addition, the M-GTA has two primary advantages over the grounded theory approach. One advantage is that M-GTA examines all interview data based on an analytic theme established by an analytical-focused individual. The selected article clarifies the focus of the analysis, which then fits within the range of actions, recognitions, emotions, and other influencing factors. Second, the M-GTA can follow something different than The Ground Theory approach, as Strauss (1987) recommended that data be broken down into small chunks, labelled, and coded. By applying the M-GTA’s coding procedures, data interpretations on an analysis worksheet would enable conceptualization directly; fine fragmentation would narrow the context of participant statements. Moreover, M-GTA offers a more practical and effective way to analyse interview data since it emphasizes the organization of substantive theories and integrates those theories with practice (Kinoshita, 2019). The M-GTA highlights actual practice, particularly in human service fields, and provides a deeper understanding of the strategy. This approach emphasizes the importance of applying practical research to alleviate existing problems and overcome knowledge polarization (Kinoshita, 2015).

### Data analysis

This study used the M-GTA (Kinoshita, 2019) to broaden the range of themes. Before coding, the study examined previous literature on refugees, COVID-19, and NGOs in both countries with the use of specific questions focusing on the impact of COVID-19 and the NGOs’ financial crisis and their impact on orphans’ health, mental health, child labour, school dropouts, educational system, and experience of trauma. The MGTA was applied using the following steps: First, the analysis began with the transcription, transcription, and translation of the first interview into English. Second, a team of three read the first interview, discussed and analysed the data, asked clarification questions, and generated theories. The next step in analysing the collected data was to categorize the data based on open coding. Using open coding, words and sentences with similar patterns were identified and integrated into categories and core categories. As the process progressed, more specific categories with similar patterns emerged, illustrating their relationships with the given concept. The following table illustrates this process using online learning challenges as an example. Then all similar sentences related to challenges were categorized under that category using codes as shown in the following example:

## Analysis worksheet of concept

**Table:** 

Concept	Challenges	Sentences
Definition	Several challenges associated with online learning during COVID-19 include internet disruptions, a lack of concentration, confusion, a lack of resources, and a lack of motivation.	
Examples	Sentence 1	*“Learning online was not suited to all children. My children do not like to learn through listening only.”*
	Sentence 2	*“She does not have a mobile phone to attend online lessons.”* *“My daughter has no tablet or laptop.”*
	Sentence 3	*“Students are more confusion and distracted during online classes.”*
	Sentence 4	*“The internet connection used to stop a lot because the network was bustling as all children had to attend online … .”* *“electronic learning wasn’t as good.”*

### Ethics and quality assurance

Before translation, the Principle Investigator randomly selected four transcribed interviews and compared the transcription with the recorded interviews. Two additional team members re-examined all translated interviews to ensure that the translation was accurate and contained the actual meaning of the content. If disagreements arose among the reach team, the team sought outside assistance from a professional translator familiar with the topic of the study.

### Recruitment

The participants were recruited through connections with leading NGOs in each country. In our initial phone call, we requested that each organization nominate one or more participants from their staff and mothers receiving services. A second call was made with the list of mothers and providers asking for their availability, followed by email confirmation and a Zoom link. Each interview lasted between 45 and 70 minutes. Before the interview, we assured all participants of confidentiality and explained the study’s primary objective. All participants agreed to begin the interview immediately after receiving more detailed answers to their questions.

### Inclusion criteria

Mother participants were screened based on the following criteria: 1) identified as Iraqi or Syrian, 2) widows who lost their husbands to war either in Iraq or Syria, 3) have at least one orphaned child, 4) receive support from one of the NGOs, and 5) are 18 years or older. NGO participants were also screened based on the following criteria: 1) they work for one of the NGOs in their country, 2) they provide services to orphans and their mothers, 3) they live in Iraq or Syria, and 4) they are 18 years or older.

This study uses the orphan definition based on Islam and tradition. Traditionally Muslims define “orphan” as a child who has lost their father, the family breadwinner (Benthall, 2019). In patriarchal societies, the loss of a mother is not viewed as disastrous. Children without mothers or absent fathers are not considered orphans by Muslim traditions and were not included in this study. The same NGOs provide support and services in both countries, each with different specializations (education, social services, financial aid, medical care, and training programmes). Some also provide humanitarian activities and programmes, including sponsoring and caring for orphans.

### Participants

There were 19 female participants in the study (11 NGO staff members and eight mothers). There are five NGO staffers and four mothers from Iraq, six NGO staff, and four from Syria. One of the Syrian NGO staff members is also a mother and was interviewed for both roles. All of the mothers lost their husbands in the war. Their ages ranged from 20 to 45 (*M* = 29.4), and they had between two and eight children (*M* = 4). All participants reported working full-time. As a result of COVID-19, many mothers lost their jobs, and some began selling home goods.

## Results

The findings from this study suggest a double jeopardy effect on refugees due to COVID-19 and the NGO financial crisis. Aside from affecting refugee health, the pandemic sent shockwaves through local and international NGOs. In addition to new concerns, orphans’ families and NGOs that serve them face old ones, increasing pressures on their health, education, economics, and social conditions. These factors can be attributed to a chain of causes interconnected by the COVID-19 lockdown and its consequences. A total of seven significant concepts were generated from the data, each of which was interrelated: worsening financial situations, deteriorating educational standards, risking physical health, harming the mental health of orphans and their mothers, increasing child labour, the rise in violence, and behavioural issues.

### Economic crisis

Definition: Most refugees and displaced people are hosted in countries with weak health, water, and sanitation systems, making them particularly vulnerable to disease. During Covid-19, many host countries experienced an economic crisis when the economy of a country or an individual deteriorated dramatically. A decline in GDP or individual income, a high unemployment rate, and a cessation of financial support typically accompanies the situation.

During the COVID-19 Pandemic, several donor countries stopped providing financial aid to NGOs. As a result, services provided by NGOs were suspended, and financial assistance decreased, affecting many refugees and orphaned children. In addition, the new measures implemented to deal with the crisis made money transfers more difficult, increasing mothers’ anxiety. According to one NGO staff: “more than 70% of families depend only on financial sponsorship, negatively affecting their economic situation and causing financial hardship” (NGO-SP#5). One of the mothers said: “We have no source of income other than the Turkish Red Crescent card, through which we pay the rent of the house and pay the debts of the supermarket every month, and we remain in debt” (SFP#4).

Syrian and Iraqi orphans rely heavily on financial sponsorship from their donors. While the subsidies were insufficient to meet the family’s basic needs, their end forced them to consider alternatives, including child labour, to survive if the orphans’ mothers lacked the necessary skills and qualifications. One mother explained her unemployment concern:

Before the COVID-19 crisis, I made and sold food at home, but now I’m out of work due to the situation. I always look for a job, and if I can’t find any, I borrow money from neighbours and relatives to meet my children’s needs

(IFB#2).

While some organizations provide food and sanitary supplies, many beneficiaries refuse to deepen their reliance on organizations and instead cling to their independence, as expressed by one of the NGO staff: “Since there are no realistic alternatives available, they become even more anxious and fearful that the financial assistance will end suddenly” (NGO-SP#14).

Some organizations assisted in addressing and supporting widows and their orphaned children, though the support was not sufficient enough, as described by one of the NGO’s staff and a mother participant:

These organizations recognized our circumstances as widows without husbands and offered their help to support us, so they paid our salaries. The salaries we receive are lower than those of Turkish people; we only earn 800 or 900 TL[about $50]

(SFP#17).

Some participants expressed the need to find more than one job. As one participant stated: “I am always forced to work more because of the difficult circumstances we live in” (SFP#6). Another participant expressed her concerns about the conflict she experienced between her job and supervision of her children’s education:

Sometimes, the teacher sends the Zoom link for the class while I go from work to home. Who should use the link to attend the lesson, me or my son? Several times, I told the teacher that the time [of the class] was not suitable

(SFP#3).

Some families tend to manage well enough as they receive support from more than one organization, as noted by one of the NGOs’ staff members:

The sisters [mothers] here have more than one sponsorship from more than one organization, so the mothers are responsible for managing their personal affairs and the affairs of their children, whether to register their children in private centres or not. Most of the mothers I dealt with managed their affairs well

(NGO-SP#17).

The same NGO staff added, describing women losing their job due to the COVID-19 lockdown:

The mothers are disappointed and unable to do anything like leave the orphanage; many wished to have extra income to rent a house independently so they can stay with their children all the time, but they have to put up with it. They are unable to leave the orphanage. I knew two women who were fired from work because of the permanent closure of the shops, and the employer fired them

(NGO-SP#17).

Similarly, two Iraqi mothers spoke of the difficult circumstances they experienced during COVID-19 and the lockdown. One participant said:

The Corona pandemic significantly affected Iraq and its foundations. There is no work anymore, and officials used to stop sending the salary from time to time. Aids did not stop but became less. The situation was challenging, and all people stayed at home jobless, some families did not receive aid, and others received help in smaller amounts than before.” (IFB#8).

Another Iraqi participant told her friend’s story:

My friend, a widow, was also affected by COVID-19 and her case was severe, so she was sick and had to use oxygen, and the doctor used to go to their home to give her a subcutaneous injection. They spent much money on that to the extent that they did not have enough money for house expenditures. All work stopped then because people were afraid to leave home. The effect of COVID in Iraq was significant and lasted for a long

(IFB#9).

A Syrian mother and NGO staff also described her situation as a refugee mother to orphaned children stating:

As mothers, we attended some courses so that we could use Zoom. Mothers with four children had a severe problem with the overlapping lesson times while only one mobile phone was available. In this case, the mother had to let every child attend their lessons for one day; this was a big problem because Coronavirus affected people suddenly, and tackling the new situation was very difficult. Also, some programs and sponsorships were delayed or stopped during the pandemic. Educational courses, for example, have completely stopped.

(NGO-SP#17).

The same participants described the situation of other families she worked with:

Corona affected the work of some families, of course, because these families used to work at home, and other people stopped coming to buy their products. The new situation affected these families badly. Some other families developed very severe illnesses. Some of them used to work in farming, but they started feeling tired and were not able to continue going to work. Corona’s time also caused obesity because of staying at home for a long time

(NGO-SP#17).

### Education and the decline in academic performance

Academic performance deteriorates for several reasons, including poor techniques and approaches that result in students losing motivation, concentration, and attendance. In addition, it is caused by structural factors such as inadequate resources and a lack of quality services. Mothers with school-age children could not purchase learning devices to help their children continue their online education. Not only did they have enough funding from NGOs, but schools were only supporting national children and not refugees, as one of the mothers explained:

I could not afford to buy my daughter a new device as I am a widow and have nothing more than my salary for livelihood, so I asked her school if they could help me with a tablet so my daughter could attend online lessons. However, tablets were given at school to Turkish students but not to Syrian refugee students

(SFP#6).

The lockdown closed many schools. Although education is the least affected sector due to citizenship and land requirements, orphan children were reported to struggle with access to primary education. As stated by a Syrian refugee mother:

My daughter was in the 5th grade when she attended a Turkish school for the first time, and her GPA was 89. During COVID-19, the school made all the students pass, even though they did not attend any classes … my daughter does not have a mobile phone to participate in online lessons **…** she passed. Currently, she is in the 7th grade, and her educational level has decreased significantly because she stopped attending school

(SFP#6).

Almost all participant mothers spoke of their children’s challenges in using the online learning system. One of the Syrian mothers explained how the lockdown generated other educational concerns, expressing:

Children were most affected due to the lockdown during Corona, and they started studying online … My children do not like to learn through listening only. They want to participate in the class, raise their hands to answer, and try to do something. Occasionally, my son left Zoom on; at other times, he did not attend Zoom classes. A few days ago, my son had 30 maths questions. I tried to help him because he missed all the necessary information in the 6th and 7th grades

(SFB#7).

Other participants put some effort into their children’s education by seeking private tutoring, as explained by one of the NGO staff members:

I enrolled my children in a private centre to improve their academic achievement, and they need extra help in addition to online lessons. Also, students are more exposed to confusion and distraction during online classes. So, students will not benefit sufficiently from these[online] lessons

(NGO-SP#18).

One NGO staff member, who is also a mother of orphan children, addressed the impact of social isolation on children’s education, noting: “As a result of the Corona pandemic, the level of education of most orphan students has decreased. Social isolation caused by the Corona pandemic has another negative impact” (NGO-SP#17). Another staff member (NGO-SP#18) elaborated on the burden and challenges children faced during the lockdown, including many enrolled in religious institutes to learn the Quran.

Several participants discussed the internet connection as another challenge that affected many children who could not use Zoom due to constant disconnections, as explained by an Iraqi mother: “The internet connection used to stop a lot because the network was bustling as all children had to attend online … online learning wasn’t as good. They didn’t focus during exams, unlike going to school.” (IFB#9).

Going back to school was another challenge many children and families faced. As one NGO staff member and a mother explained:

Spending two years out of school made it hard for many children to return. The children used to attend classes on Zoom, and a teacher came to help them keep up with their studies. After spending so much time at home, they found it extremely difficult to return to school. The first term was tough for them, but they had gotten used to the school system by the second term and followed instructions

(NGO-SP#17).

The same participants also spoke about another challenge many children and mothers faced living in a foreign country like Turkey. She stated:

Some mothers speak Turkish to help their children with their studies. Yet this can be a real problem when the mother cannot speak Turkish. The results could have been better… All the students received low marks. Also, the courses are longer, and the expenditures are expensive. For example, some students needed extra lessons but could not afford the tuition

(NGO-SP#17).

The poor quality of online learning and the challenges that orphans and their families face have led some students to drop out. One official in an organization working in Iraq stated:

There is a school dropout and a delay in studies. With the crisis, the dropout cases increased, as many orphaned families did not have the financial ability to purchase electronic devices or access the Internet. Consequently, the academic level of their children has declined. Most organizations provide supplementary education to orphans to improve their academic performance

(NGO-IP#12).

These educational activities lost their value due to quarantine and distance learning, as they were part of active learning curricula that involved students interacting with each other. Furthermore, many educational systems focus on educational aspects that do not meet orphans’ needs, do not meet their families’ aspirations, do not support the formal education curriculum, or undermine students’ knowledge and skills in scientific and social subjects. One Iraqi mother stated:

Uneducated mothers find it hard to fill this shortfall due to financial hardships and distance-learning difficulties. As such, mothers faced more pressure to follow up with their children’s education, increasing their stress levels and mental health challenges and increasing the odds of exposure to disorders

(IFP#1).

Another Iraqi mother added: “Education is the most serious problem we suffer from. There is no electronic education in my children’s schools, so I follow their lessons despite the difficulties I face in the school curricula” (IFP#2). Some mothers are forced to spend some money on private tutors; however, since this option is not available, many parents lose interest in their children’s education. Also, some mothers have psychological disorders such as depression and anxiety. Therefore, they lose interest in educating their orphaned children. According to one of the NGO staff members: “A mother’s interest [in her children’s education] is an essential factor in improving the quality of their children’s education. But mothers are no longer interested” (NGO-SP#11). An NGO staff participant affirmed this: “Distance education is useless” (NGO-SP#15).

A few participants thought of cultural issues as contributing factors to all these challenges facing orphan children. An NGO staff member said: “We provided tablets to orphans receiving our services to help them overcome obstacles such as physical isolation and the absence of students. That was insufficient since using technology is part of a cultural process” (NGO-IP#16). Another staff member added: “The current crisis has affected children’s education differently, depending on their geographical location, their cultural status, and their ability to afford and use technology” (NGO-IP#13).

### Health care issues

In addition to educational and mental health concerns, orphan children need health care. Family endangered their children’s health by forcing them to meet with other children to maintain their education. One of the NGO participants confirmed: “Some families forced their children, gathering them in one place where a computer is available through which educational materials can be displayed. Consequently, breaking social distancing rules increases the chance of infection with the pandemic” (NGO-SP#14).

One of the mothers also reported that: “They [her children] had difficulty accessing hospitals and health facilities” (SFP#3). Another NGO staff member noted: “Several parents expressed concern about not being able to conduct necessary examinations for their children or obtain the necessary medicines” (NGO-IP#12).

In addition, a mother reported, “The long stay of children at home and their extra activity sometimes put pressure on the health of mothers as well” (IFB#1). Another NGO staffer attributed some health outcomes to COVID-19 and the lockdown. She stated: “The time of corona also caused the problem of obesity because of staying at home for a long time” (NGO-SP#17).

### Mental health issues

The decline in academic achievement was not the only distance-related problem; trauma and mental health issues were also significant. The relative success of remote health care was not the same for mental health care. According to NGO participants, many mothers show a “lack of trust in mental health providers” “lack of familiarity with and availability of Telemental health services,” which has “limited many families from accessing or receiving mental health care. These issues were significant factors that exacerbated orphaned children and mothers” psychological well-being and mental health issues.

NGO staff members reported that Iraqi and Syrian refugees experience multiple psychological disorders, including “depression,” “psychological distresses,” “anxiety,” “social isolation,” “pessimistic views,” “loneliness,” “insecurity,” “emptiness,” “and loss of meaning in their lives,” and “fears of abandonment.” The psychological trauma children experience, especially those who have lost a parent, is more profound, and their needs are greater than those of other children. In schools and other educational programmes, they could release their energies, meet their peers, and mitigate and compensate for some of the losses they have suffered. One NGO staff participant said dropping out of school, and discounted education is a psychological trauma for orphans.

An NGO staff described the multiple causes affecting children’s mental health conditions:

Addressing this crisis takes a long time, as most orphans and widows suffer psychological problems (NGO-SP#10). Orphans have been significantly affected, as they have not yet recovered from the shock of war and displacement, and Corona deepened the crisis. There is nothing that we can offer them. Another NGO staff member echoed these sentiments:

“The orphans’ mental health has been greatly affected, so boredom, monotony, and depression have become part of their daily routine, as not leaving the house greatly affect them” (NGO-SP#1). Another mother stated: “My children are psychologically unstable because of the suspension of schools and the suspension of visits with neighbours, family, and friends” (IFP-#9). A mother/NGO staff participant informed the school that her child had become hyperactive during the lockdown by stating: “When my son started going to school, I told the school management that he was hyperactive and had little focus”

(NGO-SP#18).

Mothers of orphaned children developed some mental health issues. One of the mothers stated:

The COVID-19 Pandemic affected their [children’s] psychological life the most. They have developed a psychological complex about going out because the police would penalize me for that. They asked me often to take them out to a park or somewhere outside the house, but the lockdown was usually on Saturdays and Sundays, so I told my children that I could not afford to pay a fine. My daughter was severely affected. She started getting nervous about being locked at home with her brother all day while I was at work

(SPP-#6).

NGOs have suspended all their training programmes, leading to increased depression and anxiety among mothers. One NGO staff member confirmed this: “I can say that nearly 90% of the mothers of orphans need psychological support programs, which means that their psychological conditions are terrible” (NGO-SP#17). Another NGO staff explained the purpose of the training programmes:

NGOs provided these programs to help mothers access the labour market. They have experienced increased stress levels because of a lack of support to provide for themselves and their orphaned children with the essential medical materials to prevent the pandemic

(NGO-SP#14).

Iraqi and Syrian mothers have reported feeling stressed and having difficulty managing their workload and housework while keeping their children home. One of the mothers expressed gratitude to her deceased husband for his help with their children: “I used to live a comfortable life when my husband helped me out with many things, but now I have to do it all myself” (SFB#7). Another NGO member confirmed the service needs for mothers of orphaned children, stating: “Mothers need information about appropriate ways to deal with their children during this crisis” (NGO-SP#11). A mother said: “Staying at home all the time makes things more difficult, such as housework. For example, having children go out or go to school is better than staying at home” (IFB#9).

One mother reported her efforts to mitigate the impact COVID-19 has on her children and took responsibility for their psychological well-being: “To relieve their psychological stress, I take my children on trips to distant vineyards and gardens with no gathered crowds” (SFP#3).

Some humanitarian organizations provide psychological support services to Iraqi and Syrian orphaned children and their families to help them overcome the effects of war and loss. The implementation of these intervention sessions, however, requires total commitment from mothers. Thus, the support is conditional upon receiving support rather than for treatment.

### Child labor

In the wake of the COVID-19 pandemic, many NGOs lost much of their funding. As a result, the total income of families receiving financial support from NGOs severely declined. Furthermore, orphans and mothers employed before COVID-19 lost their jobs due to the lockdown. With the shift to online learning, the quality of education decreased, especially after regulations and mandatory attendance became less restricted. The new situation pushed many orphaned children to seek jobs away from home. One mother participant said: “My eldest son used to work with daily wages, and because of Corona and the ban measures, he is not allowed to go out or work” (SFB#5). One NGO staff affirmed this, asserting that “orphan labour during the current crisis has declined due to the lockdown and isolation measures” (NGO-SP#10). The family’s financial needs forced children and mothers into the job market to support their families. As one mother said: “Many children left school and started working” (IFB#8). For those children, violating isolation and emergency procedures outside their homes was permitted without severe penalties, making it easy for employers to exploit them. One of the participants reported:

My brother was in the 10th and 11th grades during Corona; he didn’t attend either of the two grades but passed. He dropped out of school and went to an area close to Antalya, where he worked to support his parents”

(SFP#6).

Many children who work and attend school spend less time on schoolwork and more time on their jobs or looking for work. The interviews revealed that some of these jobs were dangerous, disregarding existing safety precautions and risking their physical health. As one mother stated:

One of them is working at a shop. Each one is working in a different job. While working, they have fun, smoke shisha, and sit at cafés. Early labour can negatively affect a child’s development, especially if it involves dangerous tasks such as joining military groups. This is a problem and another burden for parents

(SFB#7).

Child labour is also influenced by culture, as one of the NGO’s sponsors stated: “Apart from the Corona crisis, male orphans get employed. This is the culture of the society in Iraq, and the current pause in going to school came as a new factor to increase child labour” (NGO-SP#11).

### Violence

Violence comes in many forms and shapes. It can be verbal, physical, emotional, sexual, or economic. From the early days of the COVID lockdowns, NGOs noted a significant increase in reported cases of violence between siblings and between mothers and their children. Several domestic violence cases were reported by NGO staff members rather than participant mothers due to the sensitivity, stigma, and shame around this issue.

Domestic violence between mothers and orphaned children is the most common violence reported by NGO staff. Due to their fear of losing the NGOs’ support, mothers were afraid to say violence against their children. According to the NGOs’ staff, several factors lead to increased violence, including economic insecurity, social isolation, increased contact between children and people who may suffer violence at home, and decreased reporting of violence. According to one NGO staff member: “increased violence cases have been observed, and many situations where mothers cannot deal with their children or inform them of the pandemic’s dangers. Many also complain about how hard it is for them to cope with the ongoing situation.” She added: One of the mothers wondered: “I want to know how to deal with my children during the Corona crisis” (NGO-IP#16). A mother reports that there has been violence between her children, but she could not protect them due to her work commitments. She states:

I discovered that she [daughter] beat her brother up, and he [son] ran to me to tell me what his sister did. I started yelling at her and told them I must leave the house to go to work because if I stayed home, we couldn’t find anything for a living

(SFP#6).

In addition to admitting violence between siblings, another mother blames it on the lockdown. Explaining: “During the lockdown, children under a certain age could not leave the house, making them feel bored and anxious. Yes, we experienced some of these issues” (SFP#7).

As violence among children is mainly based on NGO staff observation, an NGO staff member stated: “I noticed the unacceptable behaviour of students during online lessons, which led to confusion and distraction for others” (NGO-SP#18). Another NGO staff participant reported increased violence among children during COVID-19, stating: “Most children stayed at home for long, and this affected them negatively. There are many cases of violence among the children themselves. The Corona pandemic increased children’s problems [and] they became exposed to violence and bullying by their peers” (NGO-SP#17).

Family members also experience separation. Some NGOs worked on separating children 12 years and older from their family members to preserve cultural and religious values as mothers do share their place with other mothers and to prevent further unfavourable behaviour: One of the NGO staff/mothers stated:

In my observation, when male children reach 12 years old, they are isolated from their mothers and begin sleeping on a floor reserved for males, indicating that they are now too old to sleep on the same floor with their mothers. A mother can only see her sons during the day for two hours

(NGO-SP#18).

### Social and behavioural issues

Orphans’ educational programmes and activities were an essential space for addressing the fears they brought on by losing a parent, as well as for overcoming anxiety and depression and compensating for the loss of care and attention. As the programmes ended, various factors contributed to creating a stressful environment within the home, including the children’s playful nature and their need to express it. One of the mothers expressed this concern:

Before COVID-19, sponsors from The Family Organization would take children to amusement parks. Children stayed home during the Corona pandemic for a year and a half without being able to go to an amusement park. They stayed at home except that we sometimes visited my parents’ family and returned home

(SFP#6).

Most orphan families have no relatives in the refugee camps. Those children suffer the most, according to one of the NGO staffers:

The majority of children do not have relatives. As a result, children feel very isolated. Whenever I visit them, I notice that. Instead of meeting me, they hide in other rooms to avoid me. Due to isolation for a long time, they have lost social communication skills, especially young children severely affected

(NGO-SP#17).

Several mothers reported that their children turned to electronic devices to fill the void of social interaction. One mother stated: “My children spend most of their time using electronic devices more than before the Corona crisis” (SFP#5). Other NGO staffers spoke of the limited social interaction children and their mothers experienced after COVID-19. Some even risked going outside with their children, breaking quarantine, and ignoring social distancing rules, which threatened the whole family’s health. A few tried to guide their children away from the routine that had begun to permeate children’s lives, as told by most mothers. Some mothers express more concerns about their social isolation. One of the mothers spoke of her isolation: “I was affected at the time of Corona, but my children weren’t. I isolated myself, and it lasted for more than a month” (IFB#8). An NGO staff member affirmed the need for mothers to have time and space for themselves, saying:

Every mother must go out for refreshing and rejuvenation when her children are not home. However, during the Corona time, mothers could not leave the house; they had to stay home with their children. This problem caused me headaches. As for the social life of the orphans, we can say that they only have social relationships with their friends at school

(NGO-SP#17).

## Discussion

The COVID-19 pandemic continues to affect every aspect of people’s lives. Since 15 January 2023, the number of confirmed cases of COVID-19 has reached over 662 million and killed over 6.7 million worldwide (WHO, 2023). Studies show that COVID-19 is linked to adverse effects on people’s health, mental health, education, and socioeconomic conditions (Golberstein et al., 2020; Pokhrel & Chhetri, 2021; Ray et al., 2022; S. D. Hillis et al., 2021; Tee et al., 2020). S. D. Hillis et al. (2021), in their study of 21 countries, found that more than 113,4000 children across the globe were affected by COVID-19, and at least 1.5 million became orphans. Overall, the COVID-19 pandemic affects everyone regardless of wealth, class, or age. Although the impact of this crisis varies from nation to nation and between regions, refugees, displaced people, and orphan children are most affected, further escalating their pre-existing problems and worsening their vulnerability.

As a result of the new reality, many Iraqi and Syrian orphans and mothers face several intersecting challenges, including the financial crisis, decline in academic performance, child labour, health, and mental health issues, and social and behavioural problems, including violence. These study findings demonstrated the double-edged nature of the crisis when many nongovernmental organizations serving these populations were adversely affected by a financial crisis due to donor-country freezes (Wilke et al., 2020). Receiving countries also imposed many restrictions on NGOs to limit the spread of the pandemic. Consequently, most NGOs’ basic care programmes have been halted, including development and rehabilitation, empowerment, mental health, medical care, and education (Wilke et al., 2020).

Before COVID-19, Syrian and Iraqi orphans relied heavily on donations as a source of income, which was insufficient. The decline and discontinuity of this support exacerbated the poverty of already impoverished orphans and their mothers. Most families struggled to afford food, sanitary supplies, and school technology. In addition, formerly employed mothers lost their jobs because of lockdowns or illnesses, and many others were unable to build a better future for their orphans, leading many children to either drop out of school or join violent groups.

COVID-19, coupled with multiple displacements, cuts in financial aid, and delayed access to essential services, has adversely affected children’s access to education. Due to the spread of COVID-19, all primary and secondary schools cancelled their classes and moved to online learning. Children who live in conflicted areas, lose one or both parents, and experience family disintegration are more likely to drop out of school. Indeed, a war or conflict-prone environment is detrimental to education. According to the UNICEF report (S. D. Hillis et al., 2021), approximately 128 million primary and secondary-aged children are out of school in crisis-affected countries. For example, the conflict has affected 4.7 million children in Iraq, and 3.5 million children are out of school (UNICEF, 2021).

About 36.5 million children worldwide are displaced due to conflict and violence, and 63 million have been displaced from their schools (UNICEF, 2022). The supply declines because schools are attacked, targeted, or used for relief work, whereas teachers are killed or emigrated (Alsaid, 2019). The demand also decreases due to poverty and displacement (Al-Taee, 2011). Added to this are the high costs of education and the deterioration of the children’s mental health and physical conditions during wartime, including fear and insecurity. This problem was made worse by COVID-19 and the NGOs’ financial crisis, which forced many more orphans out of school for other related reasons. The financial pressure caused by the primary earner’s death led families to rely on their children’s labour for income. Orphans’ academic levels also declined due to health disturbances, caregivers’ low level of education, and, most importantly, the lack of support, motivation, and guidance from their working or overstressed mothers.

Syria’s pre-war education also featured high illiteracy rates and inefficient educational outputs (Gebel et al., 2012). The war has only deepened the education crisis, as one-third of its schools have been rendered unusable and more than 150,000 teachers left their job. Schools frequently became a shelter for internally displaced persons, limiting their ability to function as educational institutions and further restricting access to education (Ismael, 2019). Closures and restricted access to educational programmes disrupted orphans’ academic learning, limited their opportunities for socialization and expression, and exposed them to the threat of exploitation and violence. In addition, students have had to deal with different educational curricula, including those provided by Damascus, the Interim Administration in northern Syria, the opposition government, and the Islamic State. Between 2014 and 2017, Islamic State (ISIS) overturned the existing education system in Syria and Iraq, replacing it with an alternative programme (Arvisais & Guidere, 2020). This alternative “state program” included an inconsistent, incomplete curriculum to prepare students for military service (Wazzan, 2017).

Syrian and Iraqi children and adolescents experience exceptionally high rates of trauma from witnessing violence and fearing for their lives (Dietrich et al., 2019). Children who lose one or both parents have fewer opportunities to access education, and those who have been exposed to violence or suffered loss tend to regress in their school achievement. This trend put them in need of a quality and focused education programme that considers all the adverse effects of the traumas they have experienced.

In addition to lacking essential goods, Iraq has not been able to rebuild schools and hospitals since the 1991 Gulf War (Ismael, 2019). As a result of the dismantling of Iraq’s social infrastructure, children suffered from physical health problems, infant mortality, and a lack of educational opportunities due to endemic violence and extreme poverty (Ismael, 2019). The COVID-19 and NGO funding crises only increased pressure on the education system in these countries, making it impossible for orphans to access the care necessary to support their development and heal their trauma from war. Our findings correspond with Sherif’s (2018) study, showing that refugee and displaced children are much less likely to attend school, with approximately 60% attending primary school and 23% in lower-secondary school, compared to 90% for children and 84% for adolescents globally.

After years of war, Iraq and Syria’s education systems were overburdened by the COVID-19 pandemic and the NGOs’ financial crises. Remote learning proved to be an ineffective alternative to in-person classes in Syria and Iraq. There were several limitations to distance learning and educational technology, including lack of funding, inadequate communications infrastructure, the inability of families to afford sufficient devices, and a lack of relevant skills and knowledge (Issa & Jamil, 2010; Sirens et al., 2003). Due to schedule conflicts, limited technology access, and lack of time and knowledge among working women, mothers could only sometimes supervise and facilitate remote learning. Very few mothers could afford private tutors, though many could not. Schools continued to pass students despite absenteeism, reducing academic expectations and underprepared them for subsequent courses.

Even when children could attend classes, they struggled to stay engaged, mainly if their learning style was predominantly social or they were experiencing trauma, sickness, and other stresses at home. Educational activities in schools are often based on active learning curricula that require students to interact with each other and the tools around them. Due to quarantine and distance learning, these methods lost their value. Furthermore, many educational systems prioritize aspects of education that may not be important to orphans and may not support their mothers’ aspirations or fulfill the basic academic requirements, especially in scientific subjects. With the financial dropouts and the difficulties of distance learning, many mothers, particularly the less educated, cannot cover the educational gap for their children. The added pressure placed on mothers to follow their children’s lessons made them more likely to develop mental health challenges. Based on a modelling study (Bayham & Fenichel, 2020) of 3.1 million American individuals, school closure for children 3–13 years old may help in reducing the spread of the COVID-19 virus and save some lives; however, losing access to school healthcare workers may increase mortality so that closing schools may have a lower net benefit.

Mental health issues were prevalent among the study participants. First, war-related trauma and losses have made Iraqi and Syrian orphans particularly vulnerable to mental health issues, especially anxiety and depression. Before COVID-19, orphans received social, health, and mental health support primarily from schools, educational programmes, and other NGO-sponsored activities. Following COVID-19, many orphans lost access to stable support systems such as schools and other education programmes and activities, further deteriorating their mental health. When these programmes ceased, children experienced heightened anxiety, boredom, loneliness, isolation, depression, and more. As a result of social anxiety, many children began to fear other people and situations outside the home. The loss of primary caregivers, lack of interactions with others, and heavy use of electronic devices can severely affect orphans’ cognitive development and social communication skills. Also, children’s physical and mental health was endangered by the pressure placed on them by their mothers to congregate in ways that violated social distancing rules.

Refugees, immigrants, and asylum seekers living in insecure accommodation centres are at high risk for developing mental health issues post-COVID-19. The primary concerns are anxiety, uncertainty about the future, fear of getting sick, and facing severe financial consequences. According to the WHO (Tomczyk et al., 2021) global survey, over 50% of respondents worldwide reported more significant mental health issues, including depression, anxiety, and loneliness, with 21% using drugs or alcohol more frequently.

In addition to mental health issues, many displaced and orphaned children struggle to access health and mental health infrastructure due to bureaucratic barriers such as residency requirements (Ismael, 2019). The displacement of many orphans removed them from their usual community connections and reduced their access to social support networks. Orphans disconnected from their family and friends will most likely experience these struggles. Forced migration or displacement caused by war can interfere with healthy development and functioning, especially for youth (Dietrich et al., 2019).

Additionally, limited access to medical care, prolonged stays at home, and trauma have aggravated their health and well-being. As a result, this population’s physical and mental health problems are common, including interrupted education, developmental issues, diseases, malnutrition, and more (Lindsay et al., 2022). Orphans experience anxiety, hopelessness, and sadness due to losing their direct fatherly care. These emotions are exacerbated by experiences such as war, asylum, and loss of family members and friends (Tanga, 2013). Iraqi children’s suffering is not a new issue as it has existed since the Iraq-Iran war (1980–1988), the Gulf War (1990–1991), the United Nations sanctions on Iraq (1990), and the American war (2003) (Meberbeche & Basin, 2022). Most recently, the COVID-19 pandemic and the NGOs’ financial crisis have contributed to their suffering. The risk of psychological disorders increased during the current situation, primarily due to enforced isolation and restrictions on freedom (Cappa & Jijon, 2021; Peterman et al., 2020). Severe anxiety or depression among orphan children and their mothers can cause adverse childhood experiences and elevated levels of toxic stress. These adverse childhood experiences may increase the risk of cognitive impairment, substance abuse, depression, and non-communicable diseases in adulthood (Araújo et al., 2020).

The COVID-19 situation presents a new challenge impacting the mental health of displaced and orphan populations. Fear and uncertainty of the lockdown policy, fear of losing family members, and increased mental health issues such as anxiety, stress, depression, substance abuse, and violence against women and children are of significant concern (UNICEF, 2020). Physical distancing among this particular population is challenging, especially when many feel isolated from the rest of the country (Brooks et al., 2020).

Mothers also experienced a decline in their psychological well-being when NGOs discontinued all mental health intervention programmes. They were forced to stay home all day, caring for their children and managing the household and work. Studies confirm these findings, indicating that children and mothers during COVID-19 suffer from significant anxiety and depression (Babore et al., 2021; Gee et al., 2021; Hills et al., 2021; Meade, 2021; Rodriguez et al., 2020). SARS (2002–2004) and swine flu (2009–2010) pandemics also led to similar increases in mental health disorders and suicide attempts (Maalouf et al., 2021). Syria’s child labour problem existed before the war began, but the conflict has exacerbated it greatly (Beyer, 2012). Over 75% of households report that children work “jointly” or “solely” to provide for their families (UNICEF, 2021).

Since COVID-19, there has been a noticeable decline in labour due to a decrease in overall employment. Orphans also gained employment during these crises that intersected with several factors. Donors’ financial sponsorship ended, leaving families with no other choices. Secondly, distance education could have been more effective for most families, causing them to look for ways to invest their children’s time and energy. Employers found it easier to exploit children by violating isolation and emergency procedures because they are not subject to severe penalties. To compensate for the loss of official and legal employment, a family’s financial need may push orphans into more dangerous jobs, which may ignore existing health and safety measures.

Orphans who drop out of school and end sponsorships face complex future alternatives. The longer they stay out of school, the harder it will be for them to get back into me, and the more they work, the harder it will be for them to quit. Orphans working and in school may have to spend more time working or looking for work, leading to fewer educational opportunities as job availability increases. Seeking jobs compromises their education significantly as academic restrictions and compulsory attendance have been eased.

This culture of both societies, Iraq and Syria, and the current pause in going to school became a new factor in increasing child labour. Poor education, family pressures, and family disintegration are among other factors that motivate child labour, given that child labour, to a certain extent, is socially acceptable in Iraq and Syria. Orphans are at risk of not only leaving school but also posing a threat to their and others’ lives, especially those recruited by criminal networks. These circumstances violate orphans’ rights to healthy growth.

Disruptive or aggressive behaviour and violence between siblings and peers have increased since COVID-19, putting these children at risk for social problems. Violence between children and their mothers and between siblings increased during both crises, making them more vulnerable to violent acts. Social isolation, prolonged contact with mothers and siblings, poor mental health, and decreased reporting also exacerbate violence. In previous epidemics, violence against children and women generally increased (Peterman et al., 2020). For example, West Africa’s Ebola outbreak (2014–2016) was linked to higher rapes and physical assaults against women and girls (John et al., 2020). During the Corona pandemic crisis, violence rates tripled in Australia, Brazil, China, and the United States compared to their pre-crisis levels (Peterman et al., 2020). As Iraqi and Syrian orphans in this study are not solely affected by COVID-19, violence may evolve differently. This study highlights other potentially detrimental effects of exposure to political conflict and violence. According to the July 2022 Trafficking in Persons Report, children are still at risk of being recruited and used by multiple armed groups operating in Iraq, particularly Daesh and Iranian-backed militias (Storck, 2022). The same report also indicates that the Syrian government exploits its citizens and contributes to human trafficking crimes. Child soldiers were forced into service by the government and militias affiliated with the Syrian regime, resulting in extreme violence and retaliation from other warring parties. Due to the COVID-19 pandemic and the challenging security situation, including restricted freedom of movement, data on the current number of children affected by violence, human trafficking, and child soldiering crimes are limited. Many orphan children have also been targeted and recruited by criminal networks for illegal activities such as smuggling and oil refinery work (UNICEF, 2015). Poverty, loss of family livelihood (Lassan, 2015), being targeted for criminal activities and terrorism, and inadequate financial aid is the primary causes of child labour and violence among children in Iraq and Syria.

Conclusions from this study support existing research suggesting that orphans have adverse experiences and mental health problems that call for immediate actions by the local governments and the international communities, as well as by professional mental health providers and community-based organizations.

## Implications and limitations

This study contributes to existing research on the humanitarian crises in Syria and Iraq by integrating the COVID-19 pandemic and subsequent economic repercussions into what is already known about the region’s economic and political strife. The findings also highlighted orphans’ vulnerability to war, trauma, violence, lack of medical care, poor education, financial support, and the current COVID-19 pandemic. This study offers some implications for practice and policy based on these findings.

A more holistic approach is needed at the practice level to meet children’s psychological, educational, and health needs. It is vital for the involved NGOs and their staff members, mental health providers and social workers, and donors to assess all factors in orphans’ lives when determining any intervention service. The holistic approach also requires providers to know their clients’ intersecting circumstances. It needs to use various lenses, including the person in the environment, family dynamics, culture, politics, and structural challenges. Offering food, medical supplies, and housing are considered basic needs. However, orphans in this region need a wide range of services to treat acute and chronic physical, mental, and emotional problems. Well-trained social workers and other professional providers serving the orphan population in this region must be well-trained and equipped. They must work with healthcare providers, teachers, goods distributors, orphans, and their mothers/caretakers. A key finding from this research was that mothers’ ability and interest in their children’s education significantly impacted their child’s academic achievement during and following the lockdown.

Education is one of the most significant constraints Iraqi and Syrian orphans face. The pandemic affected many children worldwide, but these particular orphans are more likely to drop out of school, become child labourers, or be members of violent military groups. For Iraqi and Syrian orphans, access to education is crucial because school is often their only means of support through which they can access social, health, and mental services. To maintain effective NGO programmes, all fundamental needs must be integrated. Children’s decisions to drop out of school are also strongly influenced by school quality, training, and equipment. Lack of skills among staff and mothers may hinder children’s learning process. For a sustainable education system to function during both crises, it must provide infrastructure for mothers being served by orphanage organizations, training and rehabilitation for orphans and their families, and formal curriculums so orphans who fell behind academically can graduate.

Mothers and NGOs play an instrumental role in protecting children’s well-being if they receive the necessary support. Psychological stressors directly affect how orphaned mothers interact with their children. A stressed mother may struggle to understand her children’s psychological, educational, and social needs. The children might also have felt ununderstood or uncared for, which could increase their feelings of isolation and depression.

The community in which the orphan lives may pose additional risks. In addition to lacking in-person interaction with other children, friends, and family members, it is also challenging to maintain a safe environment. COVID-19, coupled with mothers’ fear of recruiting their children for military service, may increase children’s isolation, affect their mental health and well-being, and lead to depression. Furthermore, some orphans escape isolation by leaving their orphanages or engaging in risky activities. Some donors became reluctant to actively engage in securing adequate protection for these children, leaving them vulnerable. Hence, the international community should protect children’s protection rights, provide safe social and educational environments, and reduce threats to human security so that children stop suffering any long-term consequences.

This research identifies the primary concerns at the policy level and offers straightforward suggestions on what is needed. The interviews with mothers and NGO staff members provide insight into the complexities of NGO funding, including what happens when it ends abruptly. Systems should be designed so that they can continue to function when external organizations are no longer present. Given that many in this region may resist relying on NGO-provided goods and services for fear of becoming dependent, working with communities to establish more autonomous infrastructures may build trust and support from community members. The findings here illustrate that relying on NGOs for funding and social services is not a viable long-term solution: the system ceases to function when the funding disappears.

Most national and international NGOs need to be more staffed with international social workers who deliver services where needed most. To address orphans

unique needs, they must be equipped with well-trained staff and professional providers who can effectively manage them. They are hiring qualified social workers and mental health providers to advocate for the underserved in their local communities and beyond. Understanding global issues beyond borders will increase young professionals

interest in humanitarian efforts, including international social workers in Iraq and Syria or neighbouring host countries. Working with NGOs can provide young professionals with invaluable experiences, further educating and preparing them to work on global issues. This can open more job opportunities may find careers with NGOs, such as the United Nations, Save the Children, UNICEF, the World Health Organization, and Women for Women International.

Despite these profound implications, this study has some limitations. The research was conducted under various stressful conditions that may have reactivated earlier stressors among participants, which could further exaggerate the stress levels mothers and staff reported. Additionally, each group had a small sample size, which limited the results’ interpretation. All interviews were conducted via Zoom; even though Zoom provides researchers and participants with some opportunity to interact, there is the possibility of technical difficulties interrupting these communications because of poor connections, further interfering with the quality of the interviews.
